# Supplementary figures and images for: To beer or not to beer: A meta-analysis of the effects of beer consumption on cardiovascular health
Source: PLoS One. 2020 Jun 3;15(6):e0233619. doi: 10.1371/journal.pone.0233619 (PMC7269243; doi:10.1371/journal.pone.0233619)

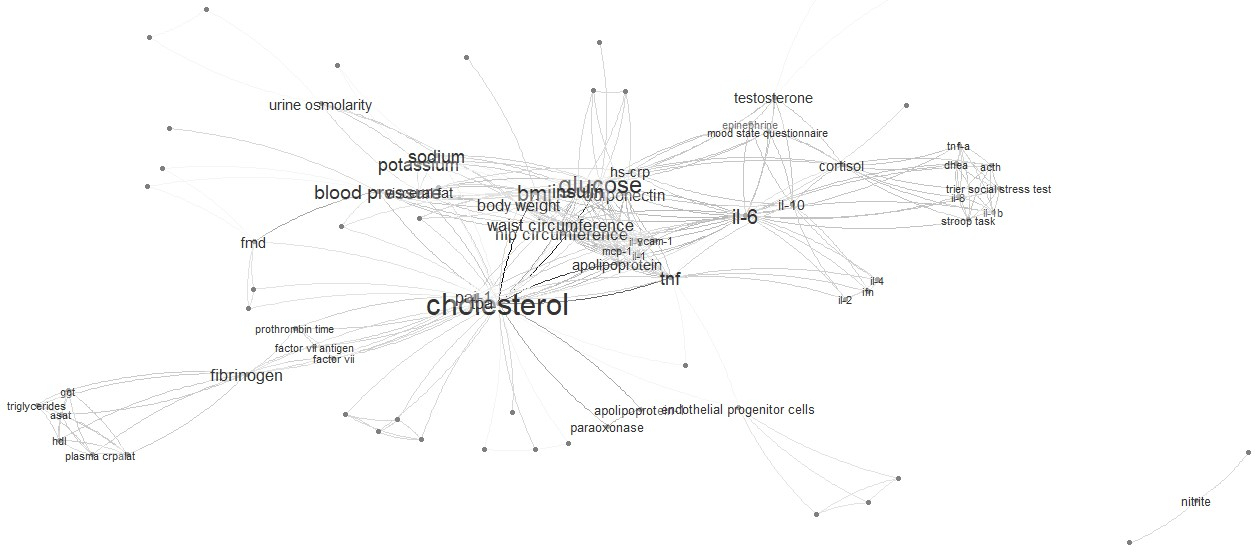

Supplement: S1 Fig — The size of the font is derived from the number of times the word is repeated. (TIF) [file pone.0233619.s001.tif]

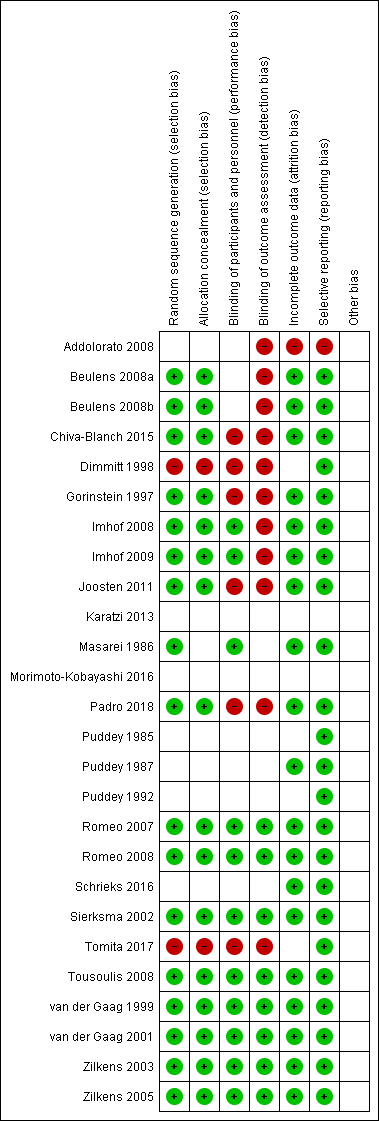

Supplement: S11 Fig — The green dot represents a low risk of bias, the red dot a high risk of bias. The lack of dot represents an intermediate risk of bias. The analysis was performed using the Cochrane risk of bias tool available at RevMan software. (TIFF) [file pone.0233619.s011.tiff]
