# Supplementary material for: To beer or not to beer: A meta-analysis of the effects of beer consumption on cardiovascular health
Source: PLoS One. 2020 Jun 3;15(6):e0233619. doi: 10.1371/journal.pone.0233619 (PMC7269243; doi:10.1371/journal.pone.0233619)
Supplement: S1 Table — (DOCX) [file pone.0233619.s012.docx]

**Supplementary table 1.** Excluded studies characteristics and exclusion reasons.

[ACTH= adrenocorticotropic hormone; ALAT= alanine aminotransferase; ASAT= aspartate aminotransferase; BMI= body mass index; DHEAS= dehydroepiandrosterone sulfate; FMD= flow mediated dilation; GGT= gamma-glutamyltransferase; HDL= high density lipoprotein; hs-CRP= high sensitivity C-reactive protein; IFN= interferon; IL-6= interleukin 6; MMP3= matrix metalloproteinase-3; MMP9= matrix metalloproteinase-9; MCP-1= monocyte chemoattractant protein-1; NA: not available; PAI-1= plasminogen activator inhibitor-1; SDP= systolic blood pressure; TMP1= tropomyosin 1; tPA= tissue plasminogen activator; TNF= tumor necrosis factor; VCAM-1= vascular cell adhesion protein 1].

| **Author** | **Journal** | **Study design** | **Intervention** | **n** | **Gender** | **Inclusion criteria** | **Aim of the study** | **Endpoints evaluated** | **Reason for exclusion** |
| --- | --- | --- | --- | --- | --- | --- | --- | --- | --- |
| Rodrigues R | Chin J Physiol. 2017 | Randomized, placebo-controlled clinical trial | Beer, 1g/kg | 10 | Male | Healthy subjects | to compare isolated and combined effects of alcohol intake and SDP on hormonal and inflammatory responses and in changes in the state of mood | Glucose, cortisol, testosterone, epinephrine, Interleukins, hs-CRP, Mood state questionnaire | Study group provides alcohol and not only beer. No results about IL-6 were available in the text |
| Benedek M | Conscious Cogn. 2017 | Placebo-controlled clinical trial | Beer, alcoholic and not-alcoholic | 70 | Both | Healthy subjects | to examine the effects of mild alcohol intoxication on creative cognition | Remote Associates Tests, Divergent Thinking Test | No data available in the results section |
| Polhuis KCMM | Nutrients. 2017 | Randomized, diet-controlled clinical trial | Beer, Wine, Spirits | 20 | Male | Older than 60 years | to investigate the diuretic effect of alcoholic beverages varying in alcohol concentration investigated the diuretic effect of alcoholic beverages varying in alcohol concentration in elderly men | Urine osmolarity | No data available in the results section |
| Quifer-Rada P | Mol Nutr Food Res. 2017 | Randomized, crossover, controlled clinical trial | Beer, alcoholic and not-alcoholic | 33 | Male | Older than 55 years and high cardiovascular risk | to study the urinary metabolomics changes | Urine metabolomics | No data available in the results section |
| Gürdeniz G | J Proteome Res. 2016 | Randomized, crossover, single-blinded clinical trial | Beer | 37 | Both | Healthy subjects | to identify the plasma and urinary metabolites associated with recent beer intake | Urine metabolomics | No data available in the results section |
| Castro-Sepulveda M | Nutrients. 2016 | Randomized, placebo-controlled clinical trial | Beer, alcoholic and not-alcoholic | 7 | Male | Soccer player | to compare the effect of beer, non-alcoholic beer and water consumption before exercise on fluid and ectrolyte homeostasis | Sodium, Potassium, Body mass | No data available in the results section |
| Rubbens J | Eur J Pharm Sci. 2016 | Cross-over, open label clinical trial | Beer, Wine, Spirits | 5 | Both | Healthy subjects | to monitor gastric and duodenal ethanol concentrations arising from the alcohol consumption | Ethanol concentration | No data available in the results section |
| Jiménez-Pavón D | J Int Soc Sports Nutr. 2015 | Cross-over, open label clinical trial | Beer | 16 | Male | Healthy subjects | to examine whether a moderate beer intake as part of the rehydration has any negative effect protocol after a short but dehydrating bout of exercise in the heat | Body weight, visceral fat, sodium, potassium, urine osmolarity | Only fat free mass measurement by DEXA |
| Petrone AB | Am J Cardiol. 2014 | Observational, clinical trial | Alcohol | 449 | Male | Heart Failure | to evaluate the mortality risk in alcohol consumers | Mortality rate, BMI | only mortality rate is reported. The alcohol administration was heterogeneous |
| Mitchell MC Jr | Alcohol Clin Exp Res. 2014 | Cross-over, open label clinical trial | Ethanol | 15 | Male | Healthy subjects | to determine the gastric emptying and absorption of ethanol | Blood Alcohol Concentration | No data available in the results section |
| Chiva-Blanch G | Atherosclerosis. 2014 | Randomized, cross-over, open label clinical trial | Beer, alcoholic and not-alcoholic | 33 | Male | High cardiovasculaar risk | to evaluate the effects of moderate beer consumption on the number of circulating EPC | Endothelial progenitor cells, MMP3, MMP9, TMP1 | only data about EPC |
| Hätönen KA | Am J Clin Nutr. 2012 | Cross-over, open label clinical trial | Beer, alcoholic and not-alcoholic | 10 | Both | Healthy subjects | to investigate the acute effects of alcohol on glucose and insulin responses and measured glycemic and insulinemic indexes | Capillary glucose and insulin response curves | No data available in the results section |
| Scherr J | Med Sci Sports Exerc. 2012 | Randomized, placebo-controlled clinical trial | Not-alcoholic beer | 277 | Male | Runners | to determine whether ingestion of not-alcoholic beer polyphenols attenuate postrace inflammation | Interleukin-6, Wisconsin Upper Respiratory Symptom Survey | the study group provided non-alcoholic beer |
| Hobson RM | Alcohol Alcohol. 2010 | Cross-over, open label clinical trial | Beer | 12 | Male | Healthy subjects | to examine the effect of alcohol on urine production | Urine volume, serum and urine osmolarity | No data available in the results section |
| Huang PH | Arterioscler Thromb Vasc Biol. 2010 | Randomized, open label clinical trial | Wine, Beer, Wodka | 80 | Both | Healthy subjects | to test the hypothesis that intake of alcohol may promote the circulating endothelial progenitor cell | Endothelial progenitor cells, TNF, cholesterol | No data available in the results section |
| Obara K | Clin Nutr. 2009 | Randomized, double-blind, placebo controlled clinical trial | Isomerized hop extract | 94 | Both | Pre-diabetes | to investigate the efficacy and safety of isohumulones for subjects with prediabetes function, inflammatory process and thrombosis/fibrinolysis system | BMI, cholesterol, glucose, insulin, sodium, potassium | No beer was provided |
| Sesso HD | Hypertension. 2008 | Observational study | Wine, Beer | 42303 | Both | Healthy subjects | to evaluate the association between alcohol intake and hypertension | hypertension, BMI, cholesterol, blood pressure | No data available in the results section |
| Shai I | Diabetes Care. 2007 | Randomized, controlled clinical trial | Wine, not-alcoholic beer | 109 | Both | Type 2 diabetes mellitus | to evaluate the effect of daily moderate alcohol intake on glycemic control in the fasting and postprandial states | fasting plasma glucose | No data available in the results section |
| Gorinstein S | Int J Food Sci Nutr. 2007 | Randomized, placebo-controlled clinical trial | Beer & antiatherosclerotic diet | 42 | Male | Hypercholesterolemia | to evaluate the antioxidant effect of polyphenol | Cholesterol, fibrinogen | No data available in the results section |
| Lukasiewicz E | Public Health Nutr. 2005 | Cross-sectional, clinical trial | Alcoholic beverages | 2691 | Both | Healthy subjects | to assess the association with waist-to-hip ratio and body mass index | Waist and hip circumferences, BMI | No data available in the results section |
| Beulens JW | Alcohol Clin Exp Res. 2005 | Randomized, diet-controlled , clinical trial | Beer, alcoholic and not-alcoholic | 19 | Both | Healthy subjects | to investigate the effect on kinetics of plasma homocysteine | Homocysteine, S-adenosyl-methionine, pyridoxal-5-phosphate | No data available in the results section |
| Sierksma A | Alcohol Clin Exp Res. 2004 | Randomized, diet-controlled clinical trial | Beer, alcoholic and not-alcoholic | 19 | Both | Healthy subjects | to examine the effect on plasma dehydroepiandrosterone sulfate testosterone, and estradiol levels | DHEAS, testosterone, estradiol | No data available in the results section |
| Sierksma A | Clin Exp Pharmacol Physiol. 2003 | Cross-sectional, clinical trial | Wine, beer | 1196 | Both | Healthy subjects | to evaluate in a predominantly wine-drinking French population whether the relation between alcohol consumption and homocysteine concentrations | serum levels of nitrate and nitrite | No data available in the results section |
| Ka T | J Rheumatol. 2003 | Randomized, diet-controlled , clinical trial | Wine, Beer, Spirits | 11 | Male | Healthy subjects | to investigate the acute and chronic effect of dinner with alcoholic beverages on serum nitric oxide metabolites | nitrate, nitrite | No data available in the results section |
| Zilkens RR | Diabetes Care. 2003 | Randomized, cross-over, open label clinical trial | Beer | 16 | Male | Healthy subjects | to determine whether reducing alcohol intake in moderate-to-heavy drinkers results in improvement in insulin sensitivity | BMI, cholesterol, glucose, insulin, HOMA index | No data available in the results section |
| Yamamoto T | Metabolism. 2002 | Open-label, clinical trial | Beer | 5 | Male | Healthy subjects | to determine whether beer increases the plasma concentration and urinary excretion of purine bases and uridine | Hypoxanthine, xanthine, and uridine | No data available in the results section |
| Sierksma A | Alcohol Clin Exp Res. 2002 | Randomized, diet-controlled, crossover, clinical trial | Beer, alcoholic and not-alcoholic | 19 | Both | Healthy subjects | to investigate the kinetics of the alcohol-induced increases in apo A-1, HDL cholesterol, and paraoxonase | Apolipoprotein A1, cholesterol, paraoxonase | No data available in the results section |
| Bleich S | Alcohol. 2001 | Open-label, clinical trial | Beer, Wine, Spirits | 60 | Both | Healthy subjects | to test the effect on cardiovascular risk factor homocysteine | homocysteine | Only data about homocysteine |
| van der Gaag MS | Eur J Clin Nutr. 2000 | Randomized, diet-controlled, cross-over study | Beer, Wine, Spirits | 12 | Male | Healthy subjects | to evaluate the in vivo effects on antioxidants, antioxidant enzymes and antioxidant capacity | serum glutathion peroxidase, erythrocyte glutathion reductase and superoxide dismutase | No data available in the results section |
| Gorinstein S | J R Soc Med. 1998 | Randomized, controlled clinical trial | Beer | 52 | Male | Healthy subjects | to investigate the effect on plasma electrolytes | sodium, potassium, cholesterol, BMI | No data available in the results section |
| Rakic V | Atherosclerosis. 1998 | Randomized, controlled clinical trial | Beer, alcoholic and not-alcoholic | 55 | Male | Healthy subjects | To determine whether the effects of drinking pattern have differential effects on serum lipids | apolipoprotein A1, cholesterol | No control group |
| Rakic V | J Hypertens. 1998 | Randomized, controlled cross-over trial | Beer | 55 | Male | Healthy subjects | to evaluate the effects of patterns of drinking on the pressor responses | blood pressure | No data available in the results section |
| Gorinstein S | J Intern Med. 1997 | Randomized, controlled clinical trial | Beer | 28 | Male | cardiovascular risk | to evaluate the influence on the status of the thrombotic activity in patients with coronary artery disease | fibrinogen, prothrombin time, coagulant activity of factor VII and factor VII antigen, PAI-1, tPA | Duplicated |
| Tomaszewski C | Ann Emerg Med. 1995 | Randomized, controlled clinical trial | Ethanol | 20 | Both | Healthy subjects | to determine the effect of acute ethanol intoxication on the results of orthostatic tilt testing. | acute tilt test | No data available in the results section |
| Hendriks HF | BMJ. 1994 | Randomized, controlled clinical trial | Wine, Beer, Spirits | 8 | Male | Healthy subjects | to evaluate the effects on the fibrinolytic system | PAI-1, tPA | Only PAI data were available |
| Burke V | Clin Sci (Lond). 1991 | Randomized, controlled clinical trial | Low-alcoholic beer | 72 | Male | Healthy subjects | to determine whether changing alcohol consumption influenced the proportion of plasma linoleic acid | linoleic acid | No data available in the results section |
| Te Wierik E | Alcohol Alcohol. 1991 | Open label clinical trial | Wine, Beer, Spirits | 8 | Both | Healthy subjects | to evaluate the additional effect on the postprandial gastrin response | gastrin | No data available in the results section |
| Cox KL | Clin Exp Pharmacol Physiol. 1990 | Randomized, controlled clinical trial | Beer | 72 | Male | Healthy subjects | to assess the combined effect of beer and physical activity | physical fitness | No data available in the results section |
| Puddey IB | Alcohol Alcohol. 1986 | Cross-sectional, clinical trial | Beer | 46 | Male | Healthy subjects | to evaluate the effect of alcoholic and low-alcoholic beers on cholesterol | cholesterol, apolipoprotein-A1 | Duplicated |
| Puddey IB | Clin Exp Pharmacol Physiol. 1985 | Randomized, controlled, crossover trial | Beer | 46 | Male | Healthy subjects | to test the pressor effect | blood pressure | Duplicated |
