# Supplementary material for: To beer or not to beer: A meta-analysis of the effects of beer consumption on cardiovascular health
Source: PLoS One. 2020 Jun 3;15(6):e0233619. doi: 10.1371/journal.pone.0233619 (PMC7269243; doi:10.1371/journal.pone.0233619)
Supplement: Attachment — Submitted filename: Rebuttal_R3_30-04-20.docx [file pone.0233619.s015.docx]

**Comments to the Author**

**Reviewer #1**

Thanks for your effort in modifying this manuscript, most of my questions have been solved. But I still have some questions and suggestions as follows:

***Comment 1***

1. *Please re-confirm your data in table 1 precisely!*

**ANSWER**: We revised the table 1.

**Comment 2**

*On the one hand, in line 108 to 111, you mentioned that “The following inclusion criteria were considered: (i) double-blind, randomized, controlled study design”, however, Ref “Zilkens RR, Burke V, Hodgson JM, et al. Red wine and beer elevate blood pressure in normotensive men. Hypertension. 2005;45(5):874–879”,” Padro T, Muñoz-García N, Vilahur G, et al. Moderate Beer Intake and Cardiovascular Health in Overweight Individuals. Nutrients. 2018;10(9):1237”, “Schrieks IC, Joosten MM, Klöpping-Ketelaars WA, Witkamp RF, Hendriks HF. Moderate alcohol consumption after a mental stressor attenuates the endocrine stress response. Alcohol. 2016;57:29–34”, “Chiva-Blanch G, Magraner E, Condines X, et al. Effects of alcohol and polyphenols from beer on atherosclerotic biomarkers in high cardiovascular risk men: a randomized feeding trial. Nutr Metab Cardiovasc Dis. 2015;25:36–45”, Karatzi K, Rontoyanni VG, Protogerou AD, et al. Acute effects of beer on endothelial function and hemodynamics: a single-blind, crossover study in healthy volunteers. Nutrition. 2013;29:1122-6.”, “Joosten MM, Witkamp RF, Hendriks HF. Alterations in total and high-molecular-weight adiponectin after 3 weeks of moderate alcohol consumption in premenopausal women. Metabolism. 2011;60:1058–1063”, “Imhof A, Plamper I, Maier S, et al. Effect of drinking on adiponectin in healthy men and women: a randomized intervention study of water, ethanol, red wine, and beer with or without alcohol. Diabetes Care. 2009;32:1101–1103” and so on are even not double-blind study.*

**ANSWER**: Thank you for your observation. This is a misprint. Indeed, we included all controlled trials, not necessarily double-blinded. We corrected the inclusion criteria part accordingly.

**Comment 2**

*On the other hand, in Ref “Tomita J, Mochizuki S, Fujimoto S, et al. Acute improvement of endothelial functions after oral ingestion of isohumulones, bitter components of beer. Biochem Biophys Res Commun. 2017;484(4):740–745”, the total number is 23, not 31.*

*In conclusion, the accuracy of the data in this article is questionable.*

**ANSWER**: We revised the manuscript, as reported in comment 1

**Comment 3**

*In line 39-40, you mentioned “In conclusion, the specific beer effect on human cardiovascular health was evaluated for the first time”, in line 329-331, you also mentioned “In conclusion, this is the first comprehensive evaluation of beer properties in the cardiovascular setting…”, however, as far as I know, there exists some researches have already evaluated the beer effect on human cardiovascular health, do you mean this paper is “the first comprehensive meta-analysis specific beer effect on human cardiovascular health”? Please clarify.*

**ANSWER**: Thank you. We agree and we corrected the discussion as you suggested.

**Comment 4**

*In table 1 and each forest plots, please add the corresponding reference number so as to assist the reader to find the reference.*

**ANSWER**: We added the references to table 1 and to forest plots.

**Reviewer #2:**

**Comment 1**

*As is clarified in the revised manuscript (page 4 lines 4-6), a moderate beer consumption was defined by the World Health Organization (WHO) as up to a can of 330 mL of beer containing about 5% w/v alcohol daily for women and up to 2 for men, which was equivalent to 16.5g/d for women and 33g/d for men. However, the levels of alcohol content were beyond this range in several studies listed in Table 1. Could they be regarded as moderate beer consumption?*

**ANSWER**: Thank you for this clarification. We considered that an average moderate beer consumption could be considered in our work. Indeed, first only some studies the daily beer administration was higher than those defined as moderate consumption, although this was a slight increase. Second, in these works, the authors themselves defined these dosages as moderate consumption. Thus, considering together these points, we could consider that on average this is a moderate beer consumption.

**Comment 2**

When it comes to the beneficial effect of beer on endothelial function raised by authors (page 2, lines 42-44, page 14, lines 286-295), the conclusion was based on the results of 4 trials, of which 2 were acute effect whereas others were chronic effect. The improvement of FMD was predominantly derived from the significant acute effect of two trials (Figure 5), which should be emphasized in conclusion. Following Reviewer 2 Comment 6, the beneficial effect of beer on endothelial function remained to be illustrated.

**ANSWER**: It is true. Probably, the beneficial effect of beer consumption on endothelial function is mainly due to acute administration, rather than chronic. We added this speculation in the discussion section as follows:

“However, this beneficial effect on endothelial function comes mainly from studies in which an acute beer administration is provided, confirming those studies in the literature in which a vascular endothelial function improvement is described after acute beer intake (41). Indeed, the acute intake could lead to the heart rate increase, the peripheral arteries dilation and the blood pressure reduction“.

**Comment 3**

On page 12, lines 246-247, the authors discussed “The effect on total cholesterol seems to be mainly related to the acute effect rather than the chronic one”, which was inconsistent with the results of sensitivity analyses, the difference between beer group and alcohol-free beer group was statistically significant (page 8, lines 172-175).

**ANSWER**: thank you for this clarification, we deleted this sentence since not supported by data.
